# Recent Advances in Cellulose Depolymerization: Mechanistic Insights, Catalytic Innovations, and Scalable Pathways for Biomass Valorization

**DOI:** 10.3390/polym18131565

**Published:** 2026-06-23

**Authors:** Marián Lehocký

**Affiliations:** Centre of Polymer Systems, Tomas Bata University in Zlín, Trida Tomase Bati 5678, 760 01 Zlín, Czech Republic; lehocky@utb.cz

**Keywords:** cellulose depolymerization, biomass valorization, lignocellulosic biorefinery, circular bioeconomy, glucose production, cellulose valorization, cellulose decomposition

## Abstract

Cellulose is the most promising abundant renewable polymer material with the highest potential for the future low-carbon biorefineries. However, its utilization in industry is limited by the structural recalcitrance as a result of organization of crystalline domains, fibrillar architecture hierarchy and intramolecular and intermolecular hydrogen bonding which is responsible for access restriction for the catalysts and consequent cleavage of the glycosidic bonds. Therefore, efficient depolymerization of cellulose is of paramount importance as a step in biomass conversion into the low molecular products. In this review, the recent advances in cellulose depolymerization are discussed. The chemical, enzymatic, thermal, thermochemical, mechanochemical, oxidative and hybrid catalytic method is thoroughly discussed. Attention is paid to the mechanism of the depolymerization reaction steps as glycosidic bond activation as hydrolytic, radical mediated, and energy assisted pathways. Selectivity and conversion efficiency based on substrate morphology, solvent system and catalyst design are also discussed. Further, there is a comparison of key performance metrics which are relevant for the industrial process as product yield, carbon efficiency, energy demand, stability of the catalyst, solvent recyclability and impact to the environmental lifecycle. The pros and cons of the various methods are also represented. Processes based on mineral acids enable rapid conversion. However, they suffer from corrosion, waste handling issues and degradation by-products. On the other hand, enzymatic depolymerization processes offer relatively high selectivity but they are limited in terms of feedstock sensitivity and slow reaction kinetics. The downstream valorization mechanisms are also described with the result being that no single available technology is capable of satisfying all industrial requirements. Thus, future progress expects integrated circular processes where advanced catalysis, process intensification and digital optimization strategies take place.

## 1. Introduction

**Figure 1:**
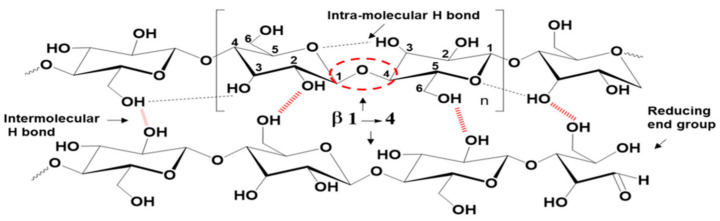
Intramolecular and intermolecular hydrogen-bonding network in cellulose illustrating the interactions responsible for the high crystallinity and structural recalcitrance of cellulose microfibrils [7].

The decarbonization of the chemical and energy industries has generated an international effort to identify sustainable sources of renewable carbon suitable for large-scale replacement of petroleum-based intermediate compounds. Lignocellulosic biomass is considered one of the best alternatives to fossil carbon as it is inexpensive, geographically dispersed, does not compete with food production, and aligns with a circular bioeconomy model. One advantage of lignocellulosic biomass over fossil carbon is that it can enter the short-term biogenic carbon cycle when managed sustainably, thus providing a route to reducing net GHG emissions associated with the use of these biomass products when combined with efficient conversion methods [1]. Of the three major components of lignocellulosic biomass (cellulose, hemicellulose, and lignin), cellulose is unique in terms of its relatively high concentration within plant cell walls, uniform structure, and ability to be converted directly into sugars, fuels, chemicals, and materials. Cellulose is produced annually at a rate greater than 100 billion tons and is therefore the largest single source of renewable, reduced-carbon material available for the development of new industrial applications, followed by chitosan and starch [2,3]. Cellulose consists of long chains of glucose molecules linked together via beta 1–4 bonds arranged in a hierarchical structure (as seen in Figure 1) consisting of crystalline and amorphous regions. Despite its relatively simple structure, cellulose has been shown to be resistant to enzymatic digestion and chemical treatment, primarily due to strong intramolecular and intermolecular hydrogen bonding, close packing of adjacent chains, the formation of aggregates called microfibrils, and associations with hemicelluloses and lignins. These interactions generate a highly recalcitrant material that has evolved to protect itself against biological and chemical attacks. Therefore, the primary barrier to converting cellulose into valuable products is not a lack of carbon availability, but rather a lack of carbon accessibility [4,5].

As such, the controlled depolymerization of cellulose is an essential first step in the operation of modern biorefineries. The breakdown of glycosidic linkages during depolymerization produces soluble oligosaccharides and monomeric sugars. They can be consequently converted, biologically or by using catalysts, to a wide range of low molecular weight products such as alcohols, organic acids, furans, polyols, sustainable aviation fuel precursors and high value fine chemicals [6].

**Scheme 1:**
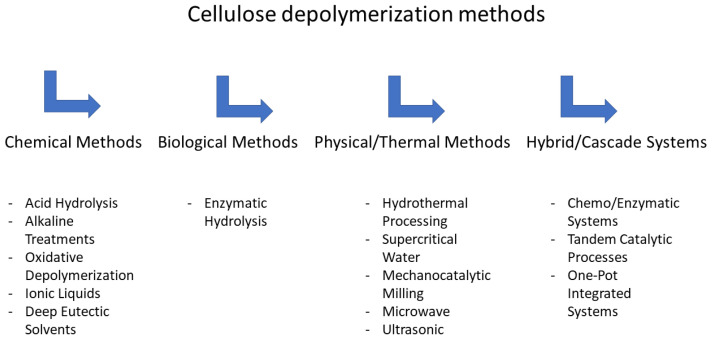
Classification of major cellulose depolymerization strategies discussed in this review, including chemical, biological, thermochemical, mechanochemical, and hybrid processing approaches.

With respect to the recent advances, there is not yet a commercially available method for efficient cellulose depolymerization which is based on environmental and economic criteria. The most promising seems to be the hydrolysis by mineral acids as it rapidly breaks glycosidic bonds but is responsible for corrosion of the equipment and degrades obtained sugars to low molecular fragments. It also requires basic neutralization of by-products which are acidic and generates wastewater. On the other hand, enzymatic hydrolysis is highly selective process and requires only mild conditions. However, the main limitations are slow heterogenous kinetics of immobilized enzymes, relative high enzyme cost, inhibition by presence of reaction byproducts and also binding to substrates containing lignin [8]. Thermochemical methods are a little different. They achieve rapid conversion but require a high amount of thermal energy. In recent years, several other methods which use ionic liquids or deep eutectic solvents which are capable to dissolve the cellulose have been recognized. Moreover, methods which use mechanical activation in order to increase the surface area of lignocellulosic substrate are interesting. Progressive methods are also electrocatalysis which lowers energy inputs, and cascadic combinations of multiple technologies [9]. In the following Scheme 1 is a classification of major cellulose depolymerization strategies discussed in this review, including chemical, biological, thermochemical, mechanochemical, and hybrid processing approaches.

A major barrier to evaluating the effectiveness of new methods for cellulose depolymerization is that most research reports their effectiveness using a limited set of narrowly defined data points. Glucose yield, reaction severity, and catalyst loadings are examples of such measures. Most studies report results under vastly different conditions (solid content, analytically determined parameters, system boundaries) than those in other studies. As such, what appear to be highly effective results from laboratory experiments are not necessarily relevant for use in industrial processes where there are many additional factors, such as: the ability to continuously circulate solvents; the need to handle solids; issues with fouling; efficiencies in recycling used solvents; the amount of water needed during processing; and maintaining continuity in processing operations [10,11,12]. The gap between optimizing a method in the laboratory and successfully implementing it in an industrial setting has slowed comparisons of technologies and hindered their commercial deployment.

There are many reviews that address the individual components of biomass pretreatment, enzymatic saccharification, solvent systems, and/or downstream upgrading individually. However, very few reviews have attempted to provide an integrated analysis of molecular recalcitrance, bond-cleavage mechanisms, catalytic platform designs, process intensification, and sustainability assessments in one place, focusing specifically on cellulose degradation. Because future advancements in these areas are unlikely to come from incremental changes to catalyst activity or yields alone, it is increasingly important that competing technologies meet all requirements for selectivity, throughput, recyclability, robustness, safety, and cost-effectiveness.

Therefore, this review aims to provide a thorough evaluation of current cellulose depolymerization technologies and strategies, including chemical, enzymatic, thermochemical, mechanochemical, oxidative, and hybrid approaches. First, the structural properties of cellulose that give rise to its recalcitrant nature and affect its reactivity are discussed. Second, the fundamental principles behind hydrolytic, oxidative and energy assisted bond cleavage are presented. Third, several of the most promising technological platforms are evaluated and compared based on key characteristics that include product yield, selectivity, energy consumption per unit product, catalyst lifetime, recoverability of solvents used in processing, environmental impact, and scalability. Fourth, possible valorization options for cellulose derived intermediate products are reviewed as part of an integrated biorefinery strategy. Finally, fifth, advances in artificial intelligence in process optimization, design of the reactors, favorable solvent systems and benchmarking protocols are discussed.

This study will explain that no single available method for cellulose depolymerization meets all requirements in terms of low energy, high conversion and environmental friendliness. Therefore, successful development of new depolymerization technologies will require decoupling structure deconstruction from selective conversion, combining catalytic and biological functionalities into one technology, and developing closed loop process architectures that optimize both material efficiency and economic viability while minimizing environmental impacts. Cellulose depolymerization, therefore, can best be thought of as not simply a hydrolysis problem but rather as a system engineering challenge that lies at the heart of creating a sustainable renewable carbon economy.

## 2. Cellulose Structure and Reactivity

**Figure 2:**
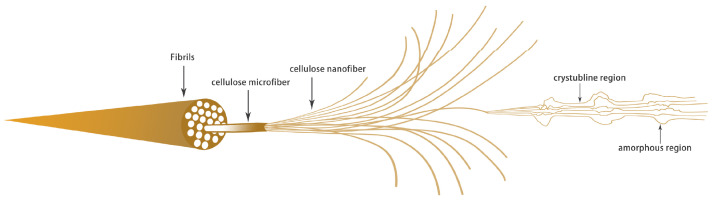
Schematic representation of the hierarchical internal structure of cellulose, showing the organization of cellulose chains into microfibrils containing crystalline and amorphous domains [15].

The simplicity of the beta-1,4-linked D-glucose monomers in cellulose contrasts sharply with the complexity of how these molecules are organized at the molecular level. Individual cellulose chains (polymers) are aligned into long, closely packed arrays, forming microfibrils [13]. These microfibrils are held together through networks of hydrogen bonding both within the molecule and between adjacent molecules. Hydrogen bonding, along with van der Waals interactions, produces areas of high crystallinity interspersed with lower-crystallinity or amorphous material [14]. Typically, each polymer has thousands of glucose units and varies based on the plant it was isolated from and the history of its purification (Figure 2). In general, the crystalline portion of the cell wall is densely packed and relatively immobile, whereas the amorphous portion is highly flexible and reactive.

The most important variables influencing cellulose reaction include porosity, moisture, associated biopolymers, and accessibility. In Table 1, there is a representation of the factors controlling the cellulose reactivity. The tight bonding (hydrogen bonding) within cellulose prevents glycoside bonds from being broken by an incoming attacking nucleophile (an atom that will be bonded to another atom). This results in the inability to dissolve cellulose in virtually all solvents, including most organic solvents, at room temperature. When dissolution can be achieved with specialized solvents, such as ionic liquids or deep eutectic solvents, the activation energy required to break the glycoside bonds remains quite high [16,17,18,19]. Water content affects hydrolysis in two ways. Low water levels enhance proton transport during acid-catalyzed hydrolysis. Excessive amounts of water can either cause recrystallization of cellulose or compete for active site(s) on the catalyst [20,21]. In addition, the rate of reactant diffusion into the porous structure of cellulose depends on both porosity and surface area. Therefore, methods for mechanically or chemically disassembling cellulose fibers (and thereby increasing porosity and surface area) generally result in higher hydrolysis rates. A major limitation to enzymatic and acid hydrolysis is the inhibitory effect of lignin and hemicelluloses present in biomass [22,23]. Hemicellulose coats individual cellulose microfibrils, creating a steric hindrance that limits the ability of enzymes or acids to contact the cellulose. Lignin acts not only as a steric hindrance to depolymerization but also has a high degree of cross-linkage and forms covalent complexes with both proteins and catalysts. These complexes effectively remove active enzyme/catalyst molecules from reacting with cellulose, thereby preventing unwanted side reactions and other problems. As these interactions between the matrix components occur with virtually no interaction with pure crystalline cellulose, this leads to exaggerated performance in laboratory experiments using pure microcrystalline cellulose [24,25].

However, crystallinity alone does not fully determine cellulose recalcitrance. An important exception to the typical relationship between cellulose crystallinity and recalcitrance is bacterial nanocellulose. Although bacterial nanocellulose possesses high crystallinity and a high degree of polymerization, it is often more susceptible to enzymatic hydrolysis than plant-derived cellulose due to its highly hydrated nanofibrillar network, large accessible surface area, high porosity, and absence of lignin and hemicellulose. These observations demonstrate that cellulose accessibility and supramolecular organization can be equally important as crystallinity in determining depolymerization efficiency.

**Figure 3:**
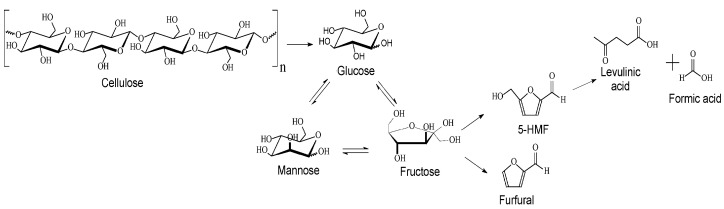
Reaction pathway illustrating cellulose hydrolysis to glucose followed by dehydration of glucose into 5-hydroxymethylfurfural (5-HMF) under acidic conditions [33].

Cellulose degradation, from both kinetic and thermodynamic perspectives, is a difficult, high-energy process that requires substantial energy to destabilize the cellulose crystal structure and break the glycosidic bonds [26]. The hydrolyzation of the 1,4 glycosidic bonds in water is favorable from thermodynamic aspects. However, the hydrolysis rate is relatively slow due to the limitations caused by diffusion and reduced number of reactive sites which are available for hydrolysis. Available kinetics studies describe so called biphase kinetics. Its first phase is relatively fast and involves the depolymerization of regions which are amorphous. In the second phase, there is kinetic deceleration of the depolymerization rate because of the predominant presence of crystalline regions [27,28]. Another important parameter is the inhibition by reaction products which slows down the reaction kinetics. This occurs when oligomers or saccharides bind and consequently accumulate on the reactive sites of enzymes and they have no more catalytic function [29,30]. Therefore, either a very acidic environment or one with a unique ability to donate protons to the glycosidic oxygen is needed for effective catalysis. However, if these extreme conditions persist for long enough, they can lead to thermodynamic runaway. Thermodynamic runaway occurs when all liberated sugars quickly dehydrate, fragment, or condense to form other thermodynamically stable species (such as levulinic acid, formic acid, or humins), rather than glucose [31,32]. Figure 3 describes the hydrolysis–dehydration reaction of cellulose to glucose and 5-HMF.

The molecular and chemical characteristics of cellulose define the development of solvents and catalysts used to degrade cellulose efficiently. Therefore, an optimal solvent system must provide sufficient solubilizing power, specifically cleave the glycosidic bonds within cellulose, and avoid excessive solvation energy required to break down cellulose into soluble components [31,34]. Additionally, all catalyst systems (homogeneous and heterogeneous) require detailed optimization of their acid-base properties, pore architecture, and surface hydrophilicity to meet the constraints of accessing cellulose. For example, solid acid catalysis may utilize mesoporous surfaces to facilitate access of large cellulose macromolecules, whereas enzyme-based catalytic systems depend on matching surface architectures and synergistically arranged active sites [35]. Further, the interactions among reaction variables, such as the relative polarities of the solvents used, the acidities of the catalysts employed, and reaction temperatures, also need to be optimized to achieve maximum depolymerization selectivity while minimizing unwanted polymerization reactions. The understanding of these interactions will be important for the future development of depolymerization technologies as more importance plays process effectivity, mild condition, high product selectivity and contamination of raw biomass by other species [36,37]. The target technology in cellulose utilization should be able to disrupt the hydrogen bonded network which is responsible for the cellulose crystalline structure formation.

## 3. Mechanisms of Cellulose Depolymerization and Classification of Depolymerization Strategies

**Figure 4:**
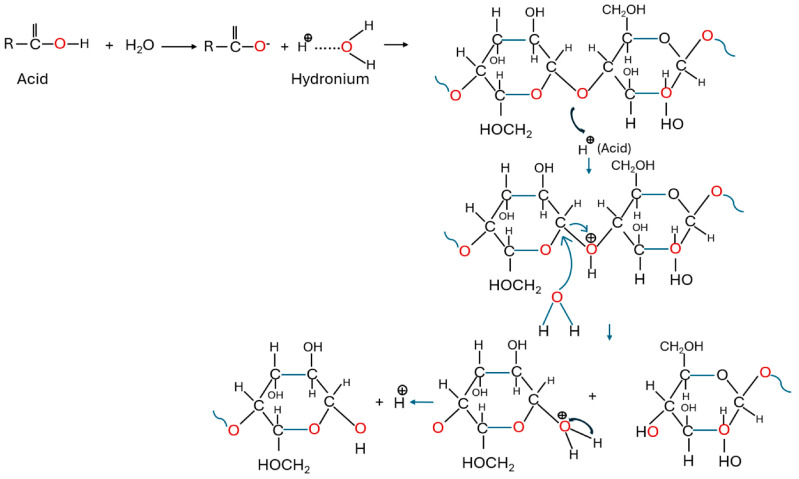
Schematic illustration of the acid-catalyzed hydrolysis mechanism of β-1,4-glycosidic bonds in cellulose, including protonation, carbocation intermediate formation, and bond cleavage [38].

The depolymerization of cellulose occurs via unique mechanisms (Figure 4) depending on factors such as solvent–catalyst interactions, the amount of energy used during depolymerization, and the environment in which depolymerization occurs.

Depolymerization through hydrolysis has been studied for many years, with hydrolysis occurring as follows: (i) a proton is placed at the glycosidic oxygen, creating a carbonium ion; and (ii) water acts as a nucleophile to create an acetaldehyde. Mineral acids have the highest concentrations of protons. Therefore, they can quickly protonate glycosidic linkages, forming a carbocation intermediate that ultimately yields either glucose or oligomers. The mechanism of this reaction is similar to that of an SN1 reaction [39,40,41]. The initial step of the reaction will be determined by how fast the carbocation intermediate forms. Although this method works well, it does lead to excessive hydrolysis. Once glucose or other oligomers are formed from cellulose, if they remain in contact with acidic environments long enough, they may dehydrate or fragment to produce smaller fragments. Comparison of acid hydrolysis methods and the parameters are mentioned in Table 2.

In addition, alkaline conditions are also suitable for cellulose depolymerization. Alkaline treatments primarily promote lignin solubilization, partial hemicellulose removal, and swelling of cellulose fibers, thereby improving cellulose accessibility to subsequent chemical or enzymatic hydrolysis. Common alkaline reagents include sodium hydroxide, calcium hydroxide, and ammonia-based systems. Alkaline cellulose treatments work under less corrosive conditions compared to mineral acids. However, alkaline wastewater generation and chemical recovery are the main problems associated with this technology and its large-scale implementation [46].

In contrast, cellulase enzymes employed in enzymatic hydrolysis use their structure binding catalytic sites. Four main types of the cellulase enzymes are available, namely, endoglucanases, exoglucanases (cellobiohydroxylases), beta-glucosidases and lytic polysaccharide monooxygenases. From these, the most progressive seems to be lytic polysaccharide monooxygenases (LPMOs) as they significantly boost the efficiency of traditional hydrolases [47,48,49]. Cellobiohydrolases remove glucose molecules from both ends of the cellulose polymer chain. Beta-glucosidases are responsible for cellobiose breakdown into the glucose [50]. Table 3 represents the main reaction parameters of key enzymes in the cellulase complex.

The synergy of these processes provides excellent specificity and enables operation at low temperature and pressure. However, there are limitations associated with enzymatic hydrolysis, including enzyme sorption/desorption kinetics and product inhibition, and limited diffusional access into crystalline structures [23].

**Figure 5:**
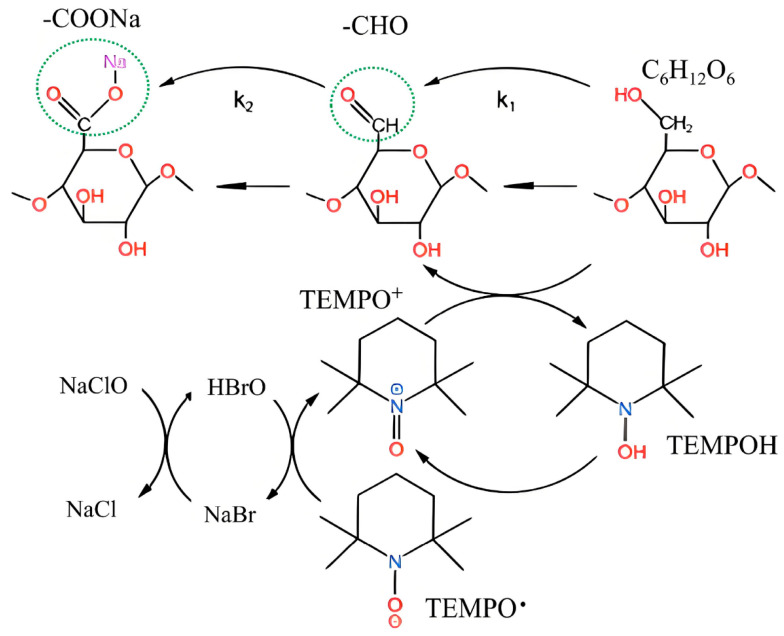
Mechanism of TEMPO-mediated selective oxidation of cellulose, showing oxidation of primary hydroxyl groups at the C6 position and disruption of cellulose crystallinity [55].

Oxidative depolymerization presents an alternative route to the conventional hydrolytic process, in which the glycosidic bond is broken using an electron transfer mechanism (or radical) to break the glycosidic bond; therefore, TEMPO-mediated oxidation (Figure 5) is a selective process that only breaks down the primary hydroxyl group at the C6 position on the cellulose chain, and thus creates swollen and thus more easily attacked cellulose chains for subsequent hydrolysis.

Although this method does not directly cleave the beta-1,4 linkage, it does reduce the degree of crystallinity and degree of polymerization, thereby facilitating further depolymerization. Metal-catalyzed oxidative cleavage of glycosidic bonds with H_2_O_2_ or molecular oxygen generates hydroxyl radicals that randomly attack glycosidic bonds, yielding a broad range of oxidatively modified fragments [56]. The metal-catalyzed oxidative cleavage of glycosidic bonds is highly dependent on reaction conditions (e.g., temperature, pH) to determine whether controlled cleavage will occur or random oxidative degradation will occur. Electrochemical and photonic catalytic processes provide an attractive means to mediate oxidative cleavage reactions and minimize the use of chemical oxidants [57].

**Figure 6:**
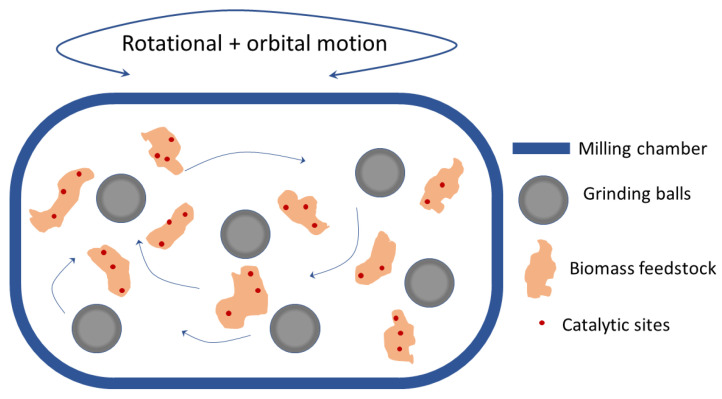
Schematic representation of planetary ball milling used for mechanocatalytic depolymerization of lignocellulosic biomass through mechanical disruption of crystalline cellulose regions.

Thermal and mechanochemical methods of degrading cellulose involve non-chemical (energetic) input rather than chemical treatment. Hydrothermal processes are methods of physically weakening hydrogen-bonded intermolecular interactions within cellulose structures by using hot water. In addition, during the process, auto-generated acids are formed from dissolved biomaterial components, which can lead to self-catalyzed hydrolysis [58,59]. Pyrolytic fast pyrolysis occurs at substantially higher temperatures than hydrothermal processing (in a low-oxygen environment), resulting in rapid thermal cleavage. As a result, fast pyrolysis produces levoglucosan and anhydro-oligosaccharides via transglycosylation mechanisms [20,60]. Mechanical depolymerization of cellulose is primarily achieved through ball milling techniques (Figure 6).

Ball milling uses shear/impact forces to mechanically break crystalline regions within cellulose, decrease particle size, and create reactive surface defects. However, when used in conjunction with solid acid catalysts, the synergy between mechanical activation (exposing glycosidic bonds) and catalysis enables the high-yield conversion of cellulose to soluble sugars [61,62,63]. The relative rate of primary bond cleavage versus secondary degradation determines product selectivity [64,65]. Therefore, optimal operating conditions require strict control over proton activity, radical flux, and/or thermal energy. Mechanistically, it has been demonstrated that understanding structural/activity relationships is key. Catalysts must possess sufficient acidity to enable activation of glycosidic bonds while possessing insufficient acidity to promote degradation of produced sugars. As to known reaction schemes for cellulose depolymerization, the depolymerization efficiency is directly proportional to the access to glycosidic bonds, the stability of intermediates as oxocarbenium ions, and the removal of sugars and oligomers from the reactive sites of enzymes. Optimized viable processes will be directly based on understanding of these parameters [29].

A new method for cellulose depolymerization employs ionic liquid-based systems. The mechanism of depolymerization is based on the disruption of hydrogen bonds between the cellulose and ionic liquid. The cellulose which is dissolved is then a subject of the mineral acid or metal catalyst hydrolyzation.

Choline chloride–urea mixtures are among the best ILs for this application due to their ability to dissolve large amounts of cellulose; however, the water content of the mixture and the amount of catalyst added need to be carefully optimized to minimize humin formation [31]. A second approach to breaking down cellulose into smaller fragments involves oxidative cleavage. This can be achieved by reacting cellulose with either oxygen, hydrogen peroxide, or electrochemically. Oxidation has several advantages over acid catalysis, since no strong acids are required [66]. However, there are also disadvantages to using oxidation to break down cellulose. For example, if too much oxygen is introduced into the reaction mixture, the resulting degradation leads to random cleavage of glycosidic bonds and subsequent fragmentation of polymer chains [67]. It should be noted that each of the above chemical methods for degrading cellulose suffers from limitations related to environmental concerns and economic feasibility. For example, the use of ionic liquids results in viscous solutions that are difficult to handle during processing and are expensive to synthesize. In addition, there are questions about whether ionic liquids are biodegradable [68,69]. Similarly, while solid acids can be readily regenerated after treatment with organic materials, they suffer from similar problems in removing adsorbed sugars or humin deposits. Further, many commercial applications of solid acids do not incorporate closed-loop solvent recovery processes, which would allow them to meet green chemistry standards [70,71]. A comparison of the most often-used solvents and catalysts for cellulose depolymerization is mentioned in Table 4.

Depolymerization of cellulose catalyzed by enzymes uses water as a solvent and a whole process run under relatively mild conditions. Each enzyme has its function. Endoglucanases break the molecule in a disordered region of the cellulose chain, while Cellobiohydrolases step-by-step release cellobiose units from the polymer chain end. Consequently, beta-glucosidase shifts depolymerization to glucose [72]. The reaction conditions are usually as pH values between 4 and 5.5 and relatively low temperatures from 40 to 50 °C. Typical is also low energy consumption and non-corrosive environment. Technology advances were recently made in terms of enzyme thermal stability, reduced ability for inhibition by reaction products and enhanced tolerance to derivatives with lignin and hemicellulose origin [73]. Another advancement was evident for cellulase enzyme immobilization onto silica mesoporous nanocrystals, magnetic particles or polymer matrix. This knowledge is important for favorable catalyst regeneration and continual process in packed-bed reactors. Despite these advances, enzyme-catalyzed hydrolysis is limited by slow kinetics, the high cost of production of cellulase enzymes, and extreme sensitivity to feedstock impurities [35,74]. Non-pressurized binding of enzymes to lignin dramatically decreases catalytic efficiency while accumulation of cellobiose and glucose reduces beta-glucosidase activity through competitive and allosteric mechanisms. The requirement for extensive pretreatment to reduce crystallinity and remove lignin adds additional complexity and capital expense to this process [20]. Therefore, the enzyme-catalyzed route provides very high selectivity and excellent environmental benefits, but poor throughput and economic competitiveness at an industrial scale without significant process intensification or reductions in manufacturing costs for enzyme production [75].

Physicochemical and thermochemical techniques use various forms of thermal energy (heat), mechanical force, and/or electromagnetic waves to break down cellulose into smaller units and to cleave chemical bonds. The primary physicochemical technique used to treat cellulose is hydrothermal treatment. This type of treatment uses elevated temperature and pressure to dissociate ions in water, resulting in an “auto-catalyst” that enables the hydrolysis of cellulose without the addition of exogenous acid. Using elevated temperatures and pressures enables an autocatalytic process [76]. Supercritical water treatment can be considered the next level up, as it operates under much more extreme conditions than those required for hydrothermal treatment. It achieves very rapid depolymerization under these conditions; however, supercritical water treatment requires specialized equipment (e.g., high-pressure reactors) and significant cooling to rapidly halt further reactions once they occur [77]. Planetary ball mills have been extensively utilized for mechanochemical treatment of biomass, including cellulose. Planetary ball milling applies extremely high shear and impact stresses to reduce crystallinity and increase the surface area of cellulose particles. As well, the planetary ball mill generates reactive free radicals on the surfaces of the treated cellulose particles. Therefore, when planetary ball milling is coupled with either a solid acid catalyst or small amounts of liquid additives, significant increases in sugar yield are observed, along with reduced time required to heat-treat the biomass [78,79]. The comparison of cellulose depolymerization methods is presented in Table 5, where the advantages and limitations of each method are also noted.

Microwave and ultrasonic-assisted depolymerization treatments provide a means to both create local areas of increased temperature (dielectric heating) that enhance mass transport and to create localized turbulence via sonic vibrations (acoustic cavitation). Both microwave and ultrasonic-assisted treatments accelerate the hydrolysis of cellulose, producing soluble oligomers and monomers [85,86]. Plasma treatments are another method used to assist in the breaking down of cellulose [87]. Plasma treatments generate reactive species, such as oxygen radicals and other oxidizing agents, that help degrade cellulose into soluble components [88,89,90].

Integrated and emerging technologies that combine multiple mechanisms to improve the performance of individual techniques include hybrid (or one-pot) systems. Mild chemical pretreatment can be used in chemo-enzymatic cascade processes to reduce the crystallinity of biomass and/or remove lignin before enzymatic hydrolysis at low temperatures for maximum yield. Acid catalysis depolymerizes biomass in tandem with isomerization or hydrogenation, enabling a single-step process in which glucose can be converted to sorbitol, mannitol, or organic acids [36,91,92]. Renewable electricity or light can power selective bond cleavage in electrocatalytic and photocatalytic depolymerization reactions, respectively. Energy inputs required for thermal degradation are minimized by using renewable energy. Reaction paths also become more predictable because they can be controlled precisely using electrical or light energy. In addition, AI/Machine Learning can be used to identify optimal catalyst formulations, predict reaction kinetic properties, and develop optimal solvents to accelerate the development of highly effective depolymerization media. Selective conversion rates are enhanced, energy requirements are reduced, and environmental sustainability is improved through lower solvent use and reduced waste generation compared with single-technique approaches [34]. However, these hybrid approaches also pose new technical challenges in process control, catalyst compatibility, and long-term operability. Successful application of hybrid technologies requires optimized integration of reaction conditions, coordination of sequential catalytic steps, and efficient separation protocols to minimize cross-inhibitory effects [93]. Thus, the best economical and environmentally friendly biorefineries model for the future is attributed to the hybrid technologies.

## 4. Performance Metrics and Comparative Analysis

Evaluating the performance of cellulose depolymerization strategies using a common evaluation method will provide a standardized means for comparing all cellulose depolymerization research based on real-world application feasibility. Although sugar yield, selectivity, and reaction severity remain widely used as general benchmarks for evaluating cellulose depolymerization research, these indicators are subject to varying interpretations due to differences in analytical methods and raw material specifications. Therefore, glucose yield alone does not accurately assess the effectiveness of many cellulose depolymerization methods, since they may be designed to produce cello-oligosaccharides or other intermediate products from cellulose conversion into platform chemicals [40,94]. Additionally, cello-oligosaccharide yields are often affected by competing reactions that produce unwanted products, including hydroxymethyl furfural, levulinic acid, and humins; not only do these undesired products decrease the economic value of the product streams, but they also make downstream processing more difficult. While reaction severity, which is most commonly defined in terms of temperature, time, and catalyst concentration, has been used to evaluate process intensities across various cellulose depolymerization methods, this variable does not account for the effects of solvents, catalyst recycling, and/or heat integration on overall system efficiency [95,96]. In addition to reaction severity and catalyst lifetime, solvent recyclability is becoming increasingly important as an additional criterion for evaluating the performance of cellulose depolymerization methods. This is particularly true for both homogeneous and heterogeneous cellulose depolymerization systems, where fouling, leaching, and changes in viscosity during operation directly affect continuous operation [97]. Several highly effective laboratory-scale catalysts have demonstrated significant losses in activity over just three to five recycle operations due to pore blockages, poisoning of active sites, or structural collapse caused by hydrothermal conditions. And finally, ionic liquids and deep eutectic solvents have positive environmental impact associated with relatively mild depolymerization reaction conditions despite their recovery require large amounts of energy for their distillation or extraction [98].

From Table 6 it is clear that there is not any single technology or depolymerization strategy which fulfills all demands on maximal process efficiency, environmental friendliness and economical rentability. Thermochemical and acid-based approaches result in high conversion but have very high energy demand and generate waste products. Oppositely, enzymatic and hybrid approaches are much more sustainable, but catalysts are relatively expensive and the whole process is more complicated in large scales. These findings emphasize that future industrial implementation will likely depend on integrated process optimization rather than maximizing individual performance metrics alone.

Assessing industrial scalability requires understanding energy consumption, mass balances, and environmental footprints. The thermochemical method requires significant thermal energy and, therefore, requires a robust heat recovery network to achieve a positive net energy balance. Low-temperature enzymatic processes require substantial energy for upstream pretreatment and downstream enzyme production. In all life cycle assessments (Table 6), it is evident that most of the environmental impact comes from producing solvents, manufacturing catalysts, and treating wastewater, usually far outweighing the reduction in carbon use from using biomass.

**Figure 7:**
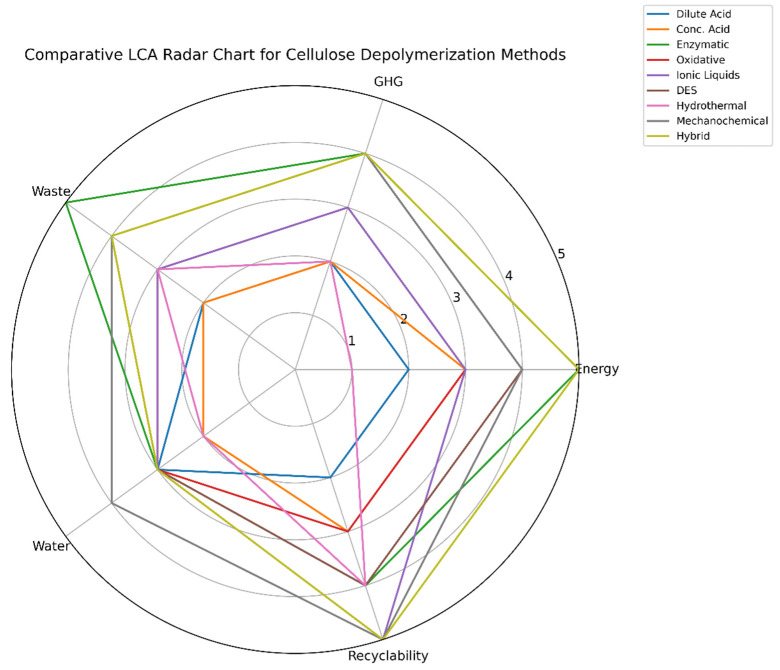
Radar chart comparing the relative life-cycle sustainability performance of major cellulose depolymerization methods based on energy demand, greenhouse gas emissions, waste generation, water consumption, and catalyst/solvent recyclability. Higher values indicate more favorable environmental performance.

From an industrial point of view it is clear that a trade-off between process sustainability and effectivity must be reached. A mineral acid system is in general very effective but produces several toxic wastes [107]. Oppositely, green solvents are environmentally friendly, but the reaction runs slowly and biocatalysts are expensive. The schematic illustration is shown on Figure 7, where there is a radar chart comparison of available methods for cellulose depolymerization. Higher scores (placed farther from the center) indicate more favorable performance in terms of environmental friendliness. The most progressive systems seem to be hybrid and enzymatic as they show the most balanced sustainability scores. On the other hand, thermochemical methods are the most energy intensive.

The distance between performing a process at lab scale versus piloting the same process is also great. Most of this is because engineers working at the bench do not consider issues such as heat transfer limitations, poor mixing, or operating continuously at larger scales when developing their ideas. Thus, standardized benchmarking protocols are urgently needed to allow comparison of different study results, including defining the system boundaries (feedstock characterization, product quantification, catalyst regeneration procedures, etc.) and which elements should be included in mass/energy balances. Without establishing common ground for comparing study results, performance data will remain fragmented, making technology transfer difficult and industry adoption nearly impossible.

Although enzymatic hydrolysis has a low environmental footprint and produces higher levels of selective conversion under milder process conditions than both acid and base catalysis, it lacks the rapid conversion kinetics that the industry has come to expect. Furthermore, the cost of purchasing enzymes is significantly greater than that of acids. However, hybrid chemoenzymatic processes are rapidly emerging as a way to take advantage of the improved selectivity of enzymes while reducing their processing energy demands and improving on solvents’ ability to reduce usage. All in all, the most important technical and economic barriers for conversion of lignocellulosic biomass are the high energy consumption before the substrate is available for enzymatic processes. Other problems are associated with solvent recovery, product separation from the reaction mixture and managing waste by-products from the depolymerization reaction.

## 5. Downstream Valorization Pathways

**Figure 8:**
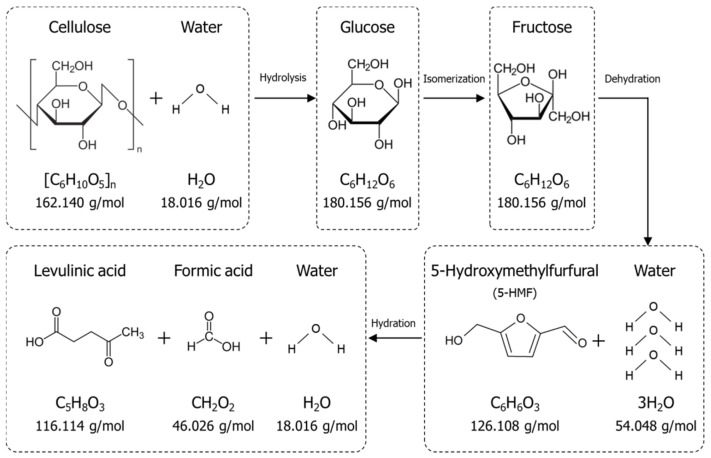
Overview of major downstream conversion pathways for cellulose-derived intermediates leading to value-added chemicals, organic acids, fuels, and functional biomaterials [114].

The products of the cellulose depolymerization reaction are available for other reactions for the final production of fuel, fine chemical reagents, and materials of high value. Their representation is shown in Figure 8. The most important product of cellulose depolymerization is glucose. Glucose is then a source for various reactions in biorefinery systems. By hydrogenation of cellulose, sugar alcohols can be produced as sorbitol or mannitol. Both are of paramount importance in pharmaceutical and food applications. Another important product is hydroxymethyl furfural, which is created by dehydration of cellulose. Hydroxymethyl furfural is a highly reactive molecule and can be converted by a hydrogenation reaction to 2,5-dimethylfuran, or by oxidation, can form 2,5-furadicarboxylic acid, which is a renewable version of terephthalic acid in polyester production [108,109]. Another important product is levulinic acid, which is formed by dehydration reactions after the isomerization of glucose to fructose. Levulinic acid is a substrate for conversion to the gamma valerolactone, alkyl levulinates, and biodegradable solvents. Cellulose can also be subjected to a retro-aldol condensation reaction leading to lower molecular-weight carbonyl compounds, such as ethylene glycol, propylene glycol or lactic acid. This demonstrates a clear possibility of cellulose replacing sources of petrochemical-derived products when carbon neutrality is maintained [110,111]. The most important product from cellulose depolymerization is ethanol, which is also produced in the largest commercial scale. This process is based on biomass fermentation. After that, the conversion to biobutanol, isobutanol, and other jet fuel precursors is possible due to the advances of metabolic engineering [112]. Another possible reaction is catalytic hydrodeoxygenation of the depolymerization byproducts, which forms renewable diesel and sustainable aviation fuel components of high energy density that are compatible with available infrastructure. However, the match between the product systems and catalytic requirements has to be reached in order to precisely control the fuel synthesis pathways. To control this step, the process also requires intermediate purification [113].

It is clear that fuels of the second generation are the main target expected from cellulose depolymerization. However, several functional materials can be prepared as cellulose-based intermediates. Cellulose nano-crystals and nano-fibrils are products of partial cellulose degradation [14]. These nanocrystals and nanofibrils are biocompatible, optically transparent, and mechanically relatively strong. Therefore, they are used in polymer matrices as reinforcing fillers or in biomedical devices and barrier film coatings [115]. Cello-oligosaccharides can be converted into carbon dots when they are subjected to an alkaline environment and heating. Such carbon dots are important due to their adjustable fluorescent properties and low toxicity, and, therefore, they are applied in bioimaging, sensor technologies, and optoelectronic applications [116]. Interesting materials can also be obtained by partial depolymerization of the cellulose derivatives. Such prepared networks and hydrogels are used in tissue engineering, drug delivery applications, and the sustainable packaging sector [117]. The downstream processing also depends on the available reaction systems and their limits. A one-pot reaction system uses a single reaction vessel for all reactions. On the contrary, a cascade reaction system contains several reaction vessels but performs multiple steps in each vessel. Both systems have reduced the isolation of intermediate products after each step. They also significantly reduce the amount of liquids and solvents used for the reaction [118]. The direct conversion of cellulose into sorbitol or mannitol is also possible with bifunctional catalysts, which have both acid and metal active sites. They are used in multistep catalysis bed reactors where the sequential dehydration/hydrogenation process produces furanic compounds [111].

Cascade approaches with thermochemical depolymerization with biological upgrading in modular biorefineries produce chemically derived sugars, which are converted into complex molecules by microorganisms. Such a hybrid approach utilizes initial resources and reduces waste byproducts [119,120].

## 6. Challenges and Limitations

Commercial-scale cellulose depolymerization process operation still needs to overcome the persistent technical and economic issues. The main problem is the recalcitrant nature of the crystalline part of the cellulose, which is the most important barrier. Two main factors have been identified; namely, an extremely dense hydrogen bonding structure and long polymer chain length. As a result, every attempt to depolymerize the cellulose molecule under relatively mild conditions is unsuccessful. Moreover, incomplete depolymerization leads to a variety of products containing unreacted cellulose, partially depolymerized or oligomeric cellulose fragments, and several mixtures of products formed during the reaction. Consequently, there is a need to separate the obtained products [121]. Another problem is the complexity of the reaction matrix in the industrial process. Real biomass varies in various crystallinity, content of lignin, mineral ash content, and moisture. The most important aspect of this is the content of lignin and hemicellulose. Lignin shields the cellulose fibril surfaces’ availability for enzymes and can be adsorbed to catalytic sites and denaturate them. Lignin can also act as an eliminator of oxygen radical species, which are responsible for breaking down the glycosidic bonds during cellulose depolymerization [122,123]. In addition, when carbohydrates present in the biomass feedstock undergo hydrolysis during processing, they produce a variety of organic acids, including acetic acid, formic acid, levulinic acid, etc., and aldehydes, including furfural, as well as other soluble compounds. When produced in sufficient quantities, these soluble compounds can lower the pH in the bioreactor to a level at which microorganisms cannot survive. Also, some metal-based catalysts can become poisoned by the accumulation of these soluble organics. Since many researchers currently use purified microcrystalline cellulose to evaluate their proposed methods for converting cellulose to biofuels, these matrix effects are commonly overlooked in laboratory studies and therefore often result in exaggerated estimates of performance that do not accurately reflect what one may expect when attempting to scale up their technology for use with raw biomass feedstocks [124].

The efficiency with which cellulose can be depolymerized greatly depends upon the type of lignocellulosic feedstock from which it is being derived. Most agricultural residues (e.g., wheat straw, corn stover) contain less lignin than other types of lignocellulosic materials; in addition, they often have a less ordered or crystalline cell wall that will allow for relatively rapid hydrolysis compared to many other feedstocks. In contrast to those feedstocks that may require some degree of processing prior to use (the so-called “pre-treatments”) hardwoods will show somewhat intermediate levels of recalcitrance (resistance to degradation by chemicals/enzymes) while their resistance to these degradative agents can vary depending upon the density of the lignin–carbohydrate complexes. Softwoods are generally the hardest lignocellulosic feedstocks for the process of depolymerization as they are comprised primarily of high levels of lignin, tightly packed structures and significant amounts of structural carbohydrates that make them resistant to both chemical and enzyme-based degradations. For this reason, feedstocks should be classified according to their lignin content, cellulose crystallinity, porosity and hemicellulose accessibility in order to determine which depolymerization strategy and pre-treatment conditions would be best suited for the selected feedstock.

All available enzymes have to be constantly replaced, which is ineffective. Mostly, it is because they are inhibited by reaction products, they degrade thermally, and also due to their affinity to lignin. Environmental and financial concerns, and high manufacturing costs, are the main problems associated with used solvents. They dissolve well but are mostly cytotoxic and non-biodegradable [34]. The reproducibility and standardization challenges are important as the feedstock is highly variable. Most of the available literature does not report methodologies for catalyst loading fully, and the process of solvent regeneration or product distribution quantification, which makes the whole process questionable in terms of technical feasibility [125].

Single processing schemes such as hybrid systems show advantages of integrated chemical and biological catalysis. For example, mild mineral acid pretreatment followed by enzymatic cellulose depolymerization shows interesting glucose yield above 80% under relatively mild reaction conditions. Mechanocatalytic systems in combination with solid acid catalysis lead to high water-soluble sugar yields with limited presence of by-products. However, persistent challenges connected with the stability of catalysts, process integration, and large-scale operation demands limit industrial implementation on a large scale.

Scale-up and process intensification present both significant engineering and economic challenges. Continuous laboratory-scale reactors cannot be relied upon to duplicate, or even approximate, the heat transfer problems, poor mixing efficiencies, and residence time distributions of continuous large-scale production systems. Exothermic reactions may lead to thermal runaway. On the other hand, endothermic reactions are subject to a reduction in conversion efficiency. Another barrier that prevents the utilization of intensified process technologies is specialized reactor requirements, corrosion-resistant materials for the reactor vessels, and expensive separation equipment. However, there are also a number of ways to address this issue through the implementation of new process intensification technologies (e.g., microwave heating, continuous-flow reactors, membrane technology), provided that sufficient pilot-plant testing and lifecycle evaluation have been conducted.

## 7. Future Perspectives and Research Directions

The future of cellulose depolymerization research will be characterized by the preparation and testing of new generations of liquids, which will be used as solvents. Another important issue that has to be addressed is connected to new catalyst preparation, as maximal recovery and environmental impact will be the most important parameters. In terms of processing liquids, their biodegradability, minimal toxicity, and easy recovery will be the main targets. Moreover, the interest in the preparation of so-called bio-eutectic and switchable ionic liquids is also increasing. Thus, all of the solutions should effectively dissolve the cellulose without any processing steps. The area of catalyst development will begin to focus on hierarchical porous solids, single-atom catalysts, and enzymatic mimetic solid structures, which incorporate the selective aspects of biological systems with the durability of heterogeneous catalysts. A hybrid approach to cellulosic depolymerization processes will become the norm as many researchers continue to develop integrated systems using AI and Machine Learning algorithms to determine the optimal catalyst–solvent combination, maximize reaction rates, and dynamically adjust process operating conditions in response to variations in feedstocks. The most important key challenges and proposed solutions are summarized in Table 7.

Circular design strategies will revolutionize biorefinery design, with an emphasis on waste-free production, closed-loop solvents & catalysts, and complete biomass fractionation. Environmentally sustainable processes will also cover the valorization of lignin and hemicellulose, besides the cellulose depolymerization, to ensure effectiveness in the use of every part of the biomass. Life-cycle assessment and other standardized methodologies will be responsible for process certifications when the full value of products is considered [126,127].

## 8. Conclusions

Cellulose depolymerization is a process of conversion of lignocellulosic materials into a variety of value-added products and in the development of biorefineries, which are sustainable. The available methods, such as chemical, enzymatic, thermal, oxidative, and mechanical, have already been intensively studied for their cellulose depolymerization capabilities. Despite the available benefits of these methods, several limitations still persist. The most important needs are environmentally friendly solvents and cost-effective and efficient production processes of the products. At this moment, still no single method meets all requirements, such as selectivity, long-term stability of used catalysts, low energy processing, solvent recycling, and environmental friendliness.

The most attractive type of technology to date appears to be the hybrid or integrated catalytic system. These types of systems provide the potential for combining the specificity associated with biological processes, and the efficiency of chemical catalysis.

However, several issues have to be addressed before the mass production in biorefineries. The first is connected to feedstock variability. The second is connected to the catalyst’s high price and its deactivation during the process. The third issue is with solvent recovery. It seems that hybrid concepts can overcome these disadvantages, but scaling up these combined systems brings additional problems. Finally, a large problem is the lack of a standardized protocol for comparison of different hybrid/combined systems.

Thus, future success in cellulose depolymerization depends on the development of stable and reasonably priced catalysts, improved pretreatment techniques, enhanced biomass fractionation processes, advancements in solvent recycling and catalyst regeneration, and more precise tools for process optimization. Valorization of cellulose biomass will be based on an engineering system that is economically viable, environmentally friendly, and sustainable.

## Figures and Tables

**Table 1:** Comparison of representative chemical, enzymatic, and hybrid cellulose depolymerization methods and their characteristic reaction features.

Factor	Problem Explanation
Crystallinity	Highly ordered crystalline regions resist chemical/enzymatic attack because tightly packed chains limit reagent penetration, reducing the availability of glycosidic bonds for hydrolysis.
Degree of polymerization	Longer chains require more bond cleavages for depolymerization, demanding harsher conditions, while chain entanglement hinders reagent diffusion to reactive sites.
Porosity	Low porosity restricts catalyst/enzyme diffusion into the cellulose matrix, creating mass transfer limitations that dominate overall reaction kinetics.
Lignin interference	Lignin physically shields cellulose and non-productively binds catalysts or enzymes, while lignin-derived compounds may inhibit catalytic activity.
Hemicellulose coating	Hemicelluloses coat cellulose microfibrils, sterically hindering access and requiring disruption before reactive agents can engage substrates.
Moisture content	Insufficient water limits cellulose swelling and reactant mobility; excess water dilutes reactants.
Catalyst accessibility	Reactive sites remain unutilized if catalysts cannot overcome steric, electrostatic, or diffusional barriers; catalyst-pore compatibility is essential.

**Table 2:** Comparison of acid hydrolysis methods.

Parameter	Dilute Acid (Less Than 5%) [42,43]	Concentrated Acid (30–70%) [44,45]
Temperature	High (160–240 °C)	Low (25–100 °C)
Glucose yield	Moderate (approx. 50–65%)	High (up to 90%)
Sugar degradation	Significant (formation HMF, furfural)	Minimal (with proper control)
Main disadvantage	High pressure and temperature required	Equipment corrosion and recovery needed

**Table 3:** Parameters of key enzymes in the cellulase complex.

Enzyme (EC Code)	Key Function (Mechanism)	Optimal pH	Optimal Temp.	Primary Product
Endoglucanases (3.2.1.4) [51]	Random cleavage within amorphous chains	4.5–5.5	45–55 °C	Short-chain polymers
Exoglucanases (3.2.1.91) [52]	Cleaving units from chain ends	4.0–5.0	50–60 °C	Cellobiose (disaccharide)
beta-glucosidases (3.2.1.21) [53]	Hydrolysis of cellobiose into monomers	4.8–5.2	45–65 °C	Glucose
LPMO (1.14.99.54) [54]	Oxidative disruption of crystalline structure	5.0–6.0	40–50 °C	Oxidized chain ends

**Table 4:** Solvents and catalysts used in cellulose depolymerization.

Solvent/Catalyst	Type	Role	Advantages	Challenges
Ionic liquids	Solvent	Dissolve cellulose	High dissolution	Expensive
Deep eutectic solvents	Solvent	Disrupt H-bond network	Green	Water sensitivity
Solid acids	Catalyst	Glycosidic cleavage	Reusable	Deactivation
Cellulases	Enzyme	Hydrolysis	Selective	Cost

**Table 5:** Comparison of most used cellulose depolymerization methods.

Method	Catalyst	Temperature	Main Products	Advantages	Limitations
Acid hydrolysis [80]	Mineral acids	120–250 °C	Glucose	Fast	Sugar degradation
Alkaline hydrolysis [46]	Alkaline reagents	60–200 °C	Carboxy acids	Equipment friendly	Low glucose yield
Enzymatic [81]	Cellulases	40–50 °C	Glucose	High selectivity	Slow kinetics
Oxidative [82]	TEMPO/metal	50–150 °C	Oxidized oligomers	Mild	Low selectivity
Hydrothermal [83]	Water	200–400 °C	Sugars	No catalyst	Energy intensive
Mechanochemical [84]	Ball milling	20–100 °C	Oligomers	No solvents	Scaling issues

**Table 6:** Life Cycle Assessment (LCA) comparison of cellulose depolymerization methods.

Method	Energy Demand	Catalyst/Solvent Impact	GHG Emissions (Relative)	Waste Generation	Water Use	Catalyst/Solvent Recyclability	Key Environmental Hotspots	Overall LCA Assessment
Dilute Acid Hydrolysis [99]	High (due to high T/P: 160–240 °C)	Mineral acids (corrosive, hazardous)	High	High (acid wastewater, degradation products like HMF, humins)	Moderate-high	Low (acid neutralization required)	Acid production, corrosion-resistant equipment, wastewater treatment	Poor-Moderate (efficient but environmentally intensive)
Concentrated Acid Hydrolysis [100]	Moderate (lower temperature)	Large acid volumes: recovery required	High	Moderate-High (acid losses, toxic residues)	High	Moderate (energy intensive acid recovery)	Acid recovery energy demand, equipment corrosion	Moderate (high yield but high environmental burden)
Enzymatic Hydrolysis [101]	Low (40–60 °C)	Biocatalysts (cellulases)	Low-Moderate	Low (minimal toxic by-products)	High (aqueous systems)	Moderate (enzyme immobilization possible)	Enzyme production, pretreatment energy, slow kinetics	Good-Very Good (green but limited by upstream impacts)
Oxidative Depolymerization (eg. TEMPO, metal catalysts) [102]	Moderate	Oxidants (H_2_O_2_, O_2_) metal catalysts	Moderate	Moderate (oxidized fragments, possible metal residues)	Moderate	Moderate	Oxidant production, catalyst leaching, selectivity issues	Moderate (balanced but selectivity-dependent)
Ionic Liquid Based Systems [103]	Moderate	IL (expensive, synthesis intensive)	Moderate-High	Low-Moderate (if recycled)	Low-Moderate	High (closed-loop possible)	IL synthesis, viscosity-related processing energy	Moderate-Good (excellent if solvent recycling is efficient)
Deep Eutectic Solvents (DES) [104]	Low-Moderate	Generally biodegradable components	Low-Moderate	Low	Moderate	Moderate-High	Water sensitivity, solvent recovery efficiency	Good (promising green alternative)
Hydrothermal Processing [20]	Very High (200–400 °C, high pressure)	Water (no added catalyst)	High	Moderate (organic acids, degraded sugars)	High	High (no catalyst needed)	Thermal energy input, pressure requirements	Moderate-Poor (simple but energy intensive)
Supercritical Water Treatment [20]	Extremely High	Water under extreme conditions	Very High	Moderate	High	High	Extreme energy input, specialized infrastructure	Poor (limited sustainability despite efficiency)
Mechanochemical (Ball Milling) [63]	Moderate (mechanical energy)	Mineral solvent use	Low-Moderate	Low	Low	High	Electricity demand, scale-up limitations	Good (low solvent footprint, scalable challenges)
Microwave/Ultrasonic Assisted [105]	Moderate	Depends on coupling chemistry	Moderate	Low-Moderate	Moderate	Moderate	Electricity source, equipment efficiency	Moderate-Good
Hybrid (Chemo–Enzymatic, Catalytic Cascades) [106]	Optimized (moderate overall)	Mixed systems (enzymes-catalysts-solvents)	Low-Moderate	Low-Moderate	Moderate	High (if integrated)	Process integration complexity, catalyst compatibility	Very Good (Best Potential)

**Table 7:** Summary of key challenges and future directions.

Challenge	Cause	Proposed Solutions
Feedstock variability	Biomass composition	Pretreatment standardization
Catalyst deactivation	Humins	Regeneration strategies
Product inhibition	Glucose accumulation	Continuous reactor
Lack of benchmarking	No standardized metrics	Universal testing protocols

## Data Availability

No new data were created or analyzed in this study. Data sharing is not applicable to this article.

## Abbreviations

The following abbreviations are used in this manuscript:

GHG emissions	Greenhouse Gas emissions
AI	artificial intelligence
EMIC	1-ethyl-3-methylimidazolium chloride
SN1	unimolecular nucleophilic substitution
HMF	hydroxymethylfurfural
TS	transition state
ET	electron transfer
IM	intermediate
LPMOs	lytic polysaccharide monooxygenases
EC Code	enzyme commission number
TEMPO	2,2,6,6-tetramethylpiperidin-1-oxyl
IL	ionic liquid
